# A Bistable Switch in Virus Dynamics Can Explain the Differences in Disease Outcome Following SIV Infections in Rhesus Macaques

**DOI:** 10.3389/fmicb.2018.01216

**Published:** 2018-06-06

**Authors:** Stanca M. Ciupe, Christopher J. Miller, Jonathan E. Forde

**Affiliations:** ^1^Department of Mathematics, Virginia Tech, Blacksburg, VA, United States; ^2^Department of Pathology, Microbiology, and Immunology, School of Veterinary Medicine, Center for Comparative Medicine and California National Primate Research Center, University of California, Davis, Davis, CA, United States; ^3^Department of Mathematics and Computer Science, Hobart and Williams Smith Colleges, Geneva, NY, United States

**Keywords:** SIV, immune complexes, mathematical model, bistable dynamics, stochastic model

## Abstract

Experimental studies have shown that the size and infectious-stage of viral inoculum influence disease outcomes in rhesus macaques infected with simian immunodeficiency virus. The possible contribution to disease outcome of antibody developed after transmission and/or present in the inoculum in free or bound form is not understood. In this study, we develop a mathematical model of virus-antibody immune complex formation and use it to predict their role in transmission and protection. The model exhibits a bistable switch between clearance and persistence states. We fitted it to temporal virus data and estimated the parameter values for free virus infectivity rate and antibody carrying capacity for which the model transitions between virus clearance and persistence when the initial conditions (in particular the ratio of immune complexes to free virus) vary. We used these results to quantify the minimum virus amount in the inoculum needed to establish persistent infections in the presence and absence of protective antibodies.

## Introduction

The humoral immune response is one of the first barriers against infecting pathogens and forms the basis for most vaccines that are currently in use (Plotkin, 2008; Deal and Balazs, 2015). The rapidly mutating human immunodeficiency virus (HIV), however, evades humoral immune responses in most human infections due to difficulties in eliciting neutralizing antibodies that are effective against the enormous diversity of virus strains (Haynes, 2015). In a few cases broadly neutralizing antibodies (bnAbs) are produced, but they appear 2–4 years following infection (Gray et al., 2011; Tomaras et al., 2011), are ineffective against co-circulating virus strains, and have unusual traits such as autoreactivity and high levels of somatic hypermutations (Mascola and Haynes, 2013). Inducing protective antibodies *in vivo* is challenging (Mascola and Haynes, 2013; Haynes, 2015), with the partially successful RV144 vaccine clinical trial offering a 31.2% decrease in transmission through non-neutralizing antibody dependent cellular toxicity-mediated responses (ADCC) (Rerks-Ngarm et al., 2009; Tomaras et al., 2013; Pollara et al., 2014).

Animal models have proven useful in examining the mechanisms of virus-antibody interactions that lead to protection against HIV infections. Studies using the chimeric simian-human rhesus macaque model (SHIV) have shown that passive transfer of broadly neutralizing monoclonal antibodies (bnMAbs) can induce protection against mucosal challenge (Moldt et al., 2012). The protection is dependent on the ratio between the challenge dose and the concentration of broadly neutralizing antibodies in the serum (Mascola et al., 1999), the breadth and potency of bnMAbs (Walker et al., 2011; Moldt et al., 2012), as well as the timing of antibody infusion (Nishimura et al., 2003). The potential for inducing neutralizing antibodies that correlate with protection *in vivo* has been shown during simian immunodeficiency virus (SIV) infections of ENV-vaccinated rhesus macaques (Letvin et al., 2011), suggesting that it may be possible to elicit antibody-mediated protection through vaccination. Understanding the properties of antibodies, such as concentration and avidity needed for protection based on known virus count in the inoculum, is important information that can guide vaccine design.

In 2009, Ma et al. used SIV infection in rhesus macaques to examine the connection between infection outcome, the size of the challenge inoculum and the disease stage in the SIV infected animals used as donors (Ma et al., 2009). They found that ~20 viral RNA (vRNA) copies titrated from a plasma pool containing virus collected during the ramp-up-stage of infection in donor animals are needed to successfully infect recipient animals. By contrast, ~1,500 vRNA copies titrated from a plasma pool containing virus collected during the set-point-stage of infection in donor animals are needed to establish infection in recipient animals. This led to the conclusion that the virus infectivity decreases over time due to a combination of virological and immunological factors. In Vaidya et al. (2010) used mathematical models to quantify the decrease in infectivity during the ramp-up and set-point infection and found that the decrease happens during both acute and chronic stages with a sharper decrease during acute infections. They did not, however, examine the mechanisms underlying the decrease.

In this study we investigate whether antiviral factors can explain the change in virus infectivity observed in experiments. Briefly, we hypothesize that donor's ramp-up-stage plasma transferred into the recipient animal contains mostly free virus. By contrast, donor's set-point-stage plasma transferred into the recipient animal contain a large amount of antibody-virus immune complexes in addition to free virus. If such immune complexes can still infect, then their infectivity rate is reduced compared to that of the free virus. To test this hypothesis, we develop a mathematical model of antibody-virus dynamics that assumes interaction between virus, recipient and donor antibody, and the corresponding immune complexes. We fit the model to viral load data from two recipient animals challenged with donor's ramp-up-stage plasma, three challenged with donor's set-point-stage plasma, and one infused with donor's set-point antibody and challenged with donor's ramp-up-stage plasma. The fits give us parameter estimates for long-run antibody concentration, free virus infectivity rates, and the relation between protection and free virus - immune complex ratio in the inoculum.

## Methods

### Data

We are using published data from the Ma et al. (2009) (all information regarding approvals by IRB can be found in the original study). Briefly, plasma samples from seven SIV infected rhesus macaques were collected during the ramp-up and set-point-stages of infections. Various amounts of vRNA were titrated from the two plasma pools and used for intravenous infection of SIV naive rhesus macaques (see Ma et al., 2009 for additional details). Animals 35036, 33815, and 3297 were challenged with virus from ramp-up-stage plasma and animals 33952, 34846, and 34373 were challenged virus from set-point-stage plasma. Lastly, animals 33681, 32350, 32970, and 36068 were challenged with virus from an aliquot of ramp-up-stage plasma containing heat inactivated set-point-stage plasma. Longitudinal virus load data (vRNA copies per ml) was collected for all recipient animals that became viremic.

### Model of recipient-virus interaction

We develop a mathematical model of virus-antibody interaction that investigates the connection between inoculum size and disease outcome. We start with the basic SIV model (Perelson et al., 1996; Bonhoeffer et al., 1997) which considers the interaction between activated uninfected CD4 T cells *T*, infected CD4 T cells *I*, and free virus *V*, as follows

(1) $$\begin{array}{r} \begin{array}{cc} \frac{dT}{dt} & =s-dT-\beta TV,\\ \frac{dI}{dt} & =\beta TV-\delta I,\\ \frac{dV}{dt} & =N\delta I-cV. \end{array} \end{array}$$

Uninfected cells are produced at rate *s*, die at per capita rate *d*, and become infected upon encountering virus at rate β. Infected cells die at per capita rate δ and produce *N* virions throughout their average lifespan. Free virus is eliminated at per capita rate *c*.

During challenge with donor's plasma, we assume that donor's antibody *A*_*D*_ and donor's virus-antibody immune complexes *X*_*D*_ are transferred into the recipient animals. *A*_*D*_ decays exponentially at rate *d*_*A*_. We assume that donor's immune complexes *X*_*D*_ can still infect target cells at rate β_1_. Their infectivity rate, however, is smaller than that of free virus, β_1_ < β. *X*_*D*_ unbind to give rise to free virus

(2) $$\begin{array}{r} X_{D}\overset{k_{m}}{\underset{k_{p}}{⇌}}V+A_{D}, \end{array}$$

where *k*_*p*_ and *k*_*m*_ are binding and unbinding rates. Lastly, *X*_*D*_ are cleared faster than free virus. We model this in a density dependent manner, with *c*_*AV*_ being the maximum removal rate and *M* the complexes at which the removal is half-maximal.

We next assume that a *de novo* antibody response to the SIV infection occurs in recipients. Recipient antibody, *A*_*R*_, binds free virus, *V*, and forms antibody-virus immune complexes, *X*_*R*_

(3) $$\begin{array}{r} A_{R}+V\overset{k_{m}}{\underset{k_{p}}{⇌}}X_{R}, \end{array}$$

with the same binding and unbinding rates as the those of the donor antibody. Recipient antibody expands in an antigen dependent manner at rate α. We account for immunological memory by assuming that recipient antibodies persist in an antigen independent manner with maximum proliferation *r* and carrying capacity *K*. Recipient immune complexes have the same infectivity rate, β_1_ < β, and the same removal rate, *c*_*AV*_>*c*, as the donor's immune complexes.

The model becomes

(4) $$\begin{array}{r} \begin{array}{cc} \frac{dT}{dt} & =s-dT-\beta TV-\beta_{1}T\left(X_{D}+X_{R}\right),\\ \frac{dI}{dt} & =\beta TV+\beta_{1}T\left(X_{D}+X_{R}\right)-\delta I,\\ \frac{dV}{dt} & =N\delta I-cV-k_{p}\left(A_{R}+A_{D}\right)V+k_{m}\left(X_{R}+X_{D}\right),\\ \frac{dA_{R}}{dt} & =\alpha A_{R}V+rA_{R}\left(1-\frac{A_{R}}{K}\right)-k_{p}A_{R}V+k_{m}X_{R},\\ \frac{dX_{R}}{dt} & =k_{p}A_{R}V-k_{m}X_{R}-c_{AV}\frac{X_{R}}{X_{R}+M},\\ \frac{dA_{D}}{dt} & =-d_{A}A_{D}-k_{p}A_{D}V+k_{m}X_{D},\\ \frac{dX_{D}}{dt} & =k_{p}A_{D}V-k_{m}X_{D}-c_{AV}\frac{X_{D}}{X_{D}+M}, \end{array} \end{array}$$

with initial values *T*(0) = *s*/*d*, *I*(0) = 0, *V*(0) = *V*_0_>0, *A*_*R*_(0) = *A*_0_, *X*_*R*_(0) = 0, *A*_*D*_(0)≥*A*_0_ and *X*_*D*_(0)≥0.

### Parameter values and initial conditions

We assume that we have *T*(0) = 10^6^ per ml and *I*(0) = 0 per ml at the beginning of infection. Uninfected CD4 T cells are produced at rate *s* = 10^4^ per ml per day (Sachsenberg et al., 1998) and die at rate *d* = 0.01 per day (Stafford et al., 2000). We use previous estimates for the infected cells death rate, δ = 0.39 per day (Markowitz et al., 2003), virus clearance rate, *c* = 23 per day (Ramratnam et al., 1999), and virus production by an infected cells, *N* = 2, 000 per day (Ciupe, 2015). We fix the immune complexes infectivity rate $\beta_{1}=10^{-8}$ ml per day per virion.

Since all donor and recipient animals were negative for anti-SIV antibodies, we assume the antibodies are below their limit of detection of 3.8 × 10^8^ molecules per ml (0.1 ng/ml^1^). Without loss of generality, $A_{D}\left(0\right)=A_{R}\left(0\right)=3.5\times 10^{8}$ molecules per ml. The recipient immune complexes are absent at the time of infection, *X*_*R*_(0) = 0 molecules per ml. Donor antibodies decay at rate *d*_*A*_ = 0.07 per day (Zalevsky et al., 2010). Once infection occurs, recipient antibodies are produced and expand in both virus-dependent and virus-independent manners, at rates α and *r*, respectively. Since α and *r* have complementary functions (see Supplementary Material), we can ignore one of them. For simplicity, we set α = 0. Immune complexes dissociate at rate *k*_*m*_ = 100 per day (Schwesinger et al., 2000; Zhou et al., 2007; Tabei et al., 2012). The IgG affinity *K*_*A*_ = *k*_*p*_/*k*_*m*_ in a humoral response frequently starts at 10^5^ M^−1^ (Gopalakrishnan and Karush, 1975). For SIV, each virion can have ten to hundreds of potential antibody binding sites and affinity maturation may occur. Taking both effects into account can increase the functional affinity *K*_*A*_ to 10^8^ M^−1^. Therefore, we consider a binding rate $k_{p}=K_{A}\times k_{m}=10^{10}M^{-1}$/day = 1.6 × 10^−11^ ml per molecule per day, higher than in Tabei et al. (2012), Ciupe (2015) and a carrying capacity *K* = 5 × 10^13^ molecules per ml. We assume that a maximum of $c_{AV}=10^{6}$ immune complexes are removed per day, and that the removal rate is half-maximal for *M* = 500 immune complexes.

When an animal is challenged with SIV, the inoculum plasma may contain both free virus or donor's immune complexes. As in Vaidya et al. (2010), we assume that the initial virus distributes throughout the entire plasma volume of a 7 kg macaque, approximately 300 ml. Therefore, our initial conditions are *X*_*D*_(0)+*V*(0) = *D*(0)/300 copies per ml, where *D*(0) is the inoculum vRNA (Ma et al., 2009), and *V*(0) and *X*_*D*_(0) vary.

We assume that the free virus infectivity rate β(>β_1_) ml per day per virion, antibody carrying capacity *K* molecules per ml, and antibody independent expansion rate *r* per day are unknown and we estimate them through data fitting. All fixed parameters and initial conditions are presented in Table 1.

**Table 1:** Parameter values and initial conditions used in model (6).

**Variables**	**Description**	**Initial values**
*T*	Target cells (cells ml^−1^)	*T*(0) = 10^6^
*I*	Infected cells (cells ml^−1^)	*I*(0) = 0
*V*	Free virus (virion ml^−1^)	*V*(0) varies
*A*_*D*_	Donor antibody (molecules ml^−1^)	*A*_*D*_(0) varies
*A*_*R*_	Recipient antibody (molecules ml^−1^)	$A_{H}\left(0\right)=3.5\times 10^{8}$
*X*_*D*_	Donor immune complexes (complexes ml^−1^)	*H*_*D*_(0) varies
*X*_*R*_	Recipient immune complexes (complexes ml^−1^)	*H*_*X*_(0) = 0
**Parameters**	**Description**	**Values**
*s*	CD4 T cell production rate (cells ml-day^−1^)	10^4^
*d*	Target CD4 T cells death rate (day^−1^)	0.01
β	Free virus infectivity rate (ml day-virion^−1^)	estimated
β_1_	Immune complexes infectivity rate (ml day-virion^−1^)	10^−8^
δ	Infected cells death rate (day^−1^)	0.39
*N*	Burst size (virion)	2000
*c*	Virus clearance rate (day^−1^)	23
α	Antigen dependent expansion of antibodies (ml day-virion^−1^)	10^−9^
*r*	Antibody division rate (day^−1^)	estimated
*d*_*A*_	Antibody degradation rate(day^−1^)	0.07
*K*	Antibody carrying capacity (molecules ml^−1^)	5 × 10^13^
*k*_*p*_	Binding rate (ml day-virion^−1^)	1.6 × 10^−11^
*k*_*m*_	Unbinding rate (day^−1^)	100
*c*_*AV*_	Immune complexes clearance rate (complexes day^−1^)	10^6^
*M*	Immune complexes where clearance is half maximal (ml^−1^)	500

### Data fitting

We estimate parameters β and *r* by simultaneously fitting *V*_*T*_(*t*) = *V*(*t*)+*X*_*D*_(*t*)+*X*_*R*_(*t*) given by model (6) with known total virus initial conditions to both chronic and no-infection virus data. Two monkeys (35036 and 33815) were protected when challenged with 2 ramp-up vRNA and chronically infected when challenged with 20 ramp-up vRNA. We use these to get initial concentrations of *V*_*T*_(0) = 2/300 and *V*_*T*_(0) = 20/300 copies per ml, respectively. When a monkey is protected, no vRNA data is collected. We create an artificial data set *VTiter*_*low*_ = {2/300, 1/300} copies per ml at times τ_*j*_∈{0, 30} days in each animal that did not get infected. When a monkey gets infected, total virus concentrations *VTiter*_*high*_(*t*_*i*_) above the limit of detection were collected at *t*_*i*_∈{2, 5, 7, 9, 12, 14, 21, 28} days post infection for each subject.

Similarly, three monkeys (33952, 34846, and 34373) were protected when challenged with 1.5, 15, and 150 set-point vRNA and chronically infected when challenged with 1500 set-point vRNA. We therefore use initial concentrations *V*_*T*_(0) = 150/300 and *V*_*T*_(0) = 1, 500/300 copies per ml for *V*_*T*_(0) in model (6). When a monkey is protected, no vRNA data is collected. We create an artificial data set *VTiter*_*low*_ = {150/300, 1/300} copies per ml at times τ_*j*_∈{0, 30} days in each animal that did not get infected. When a monkey gets infected, total virus concentrations *VTiter*_*high*_(*t*_*i*_) above the limit of detection were collected at *t*_*i*_∈{5, 7, 9, 12, 14, 21, 28} days post infection for subjects 33952 and 34373, and $t_{i}\in \bar{t}=\left\{14,21,28\right\}$ days post infection for subject 34846.

We use the “fminsearch” algorithm in MATLAB R2016b [The MathWorks Inc., Natick, MA] to minimize the functional

(5) $$\begin{array}{l} J(\beta ,K,r)=(\sum_{i=1}^{n}{\left(\log V_{T}(t_{i})-\log VTiter_{high}(t_{i})\right)}^{2}\\ \;{+\sum_{j=1}^{2}{\left(\log V_{T}(\tau_{j})-\log VTiter_{low}(\tau_{j})\right)}^{2})}^{1/2}, \end{array}$$

where *n* is the number of data points. Finally, *V*_*T*_(*t*_*i*_) and *V*_*T*_(τ_*j*_) are the theoretical predictions for the total viral concentration as given by model (6) at times *t*_*i*_ and τ_*j*_.

## Results

### Antibody-dependent basic reproduction number

The model exhibits bistable switch between a clearance state *S*_0_ = (*s*/*d*, 0, 0, *K*, 0, 0, 0) and a positive chronic state *S*_1_ = (*T*_1_, *I*_1_, *V*_1_, *A*_*R*1_, *X*_*R*1_, 0, 0). We can show analytically that steady state *S*_0_ is locally asymptotically stable when $R_{0}^{a}<1$ (see Supplementary Material), where

(6) $$\begin{array}{r} R_{0}^{a}=R_{0}\frac{k_{m}+\frac{c_{AV}}{M}+\frac{\beta_{1}}{\beta }k_{p}K}{k_{m}+\frac{c_{AV}}{M}+\frac{c_{AV}}{cM}k_{p}K}<1. \end{array}$$

Inequality (8) shows that even if viremia occurs in the absence of antibodies, $R_{0}=\frac{N\beta s}{dc}>1$, viral clearance can be reached when the combined contribution of the protective and/or infused antibodies make $R_{0}^{a}<1$. We name $R_{0}^{a}$, the antibody-dependent basic reproduction number, which represents the number of virion produced in average by an infected cell in an otherwise infection-free population throughout its lifetime when antibodies are present.

$R_{0}^{a}<1$ is a necessary but not sufficient condition for virus clearance. Indeed, numerical results show that for $R_{0}^{a}<1$, the chronic state *S*_1_ is asymptotically stable as well. That means that given $R_{0}^{a}<1$ and appropriate initial conditions the chronic steady state *S*_1_ can be reached. We numerically investigate the relationship between the model's initial conditions, animal data and the model's long-term behavior.

### Infection with ramp-up-stage virus

Animals 35036 and 33815 were challenged with 20 vRNA copies that were titrated from a plasma pool containing virus collected from seven monkeys during the ramp-up-stage of infection (defined as approximately 7 days after challenge). Following challenge they became viremic. In contrast, animal 32970, who was challenged 2 vRNA copies from the same plasma pool, did not develop a persistent infection (Ma et al., 2009). Since immune complexes are detected around 21 days post infection (Tomaras et al., 2008; Liu et al., 2011), we assume that the ramp-up inoculum contains only free virus. Therefore, our initial concentrations are *X*_*D*_(0) = 0 and *V*(0) = 20/300 copies per ml in animals 35036 and 33815; *V*(0) = 2/300 copies per ml in animal 32970. The best estimates for β and *r* were obtained by minimizing *J* given by (7) for the above initial conditions (see Table 2).

**Table 2:** Parameter estimates and 95% confidence intervals from minimizing the likelihood function *J* given by (7) for the ramp-up data.

**Animal**	**β (ml day-virion^−1^)**	**95*%* CI**	***r*(day^−1^)**	**95*%* CI**	**$R_{0}^{a}$**
35036	1.58 × 10^−7^	[1.57 × 10^−7^, 1.59 × 10^−7^]	2.32	[2.3, 2.33]	0.42
33815	1.34 × 10^−7^	[1.33 × 10^−7^, 1.35 × 10^−7^]	1.95	[1.945, 1.952]	0.35
Median	1.46 × 10^−7^	–	2.14	–	–

**Figure 1:**
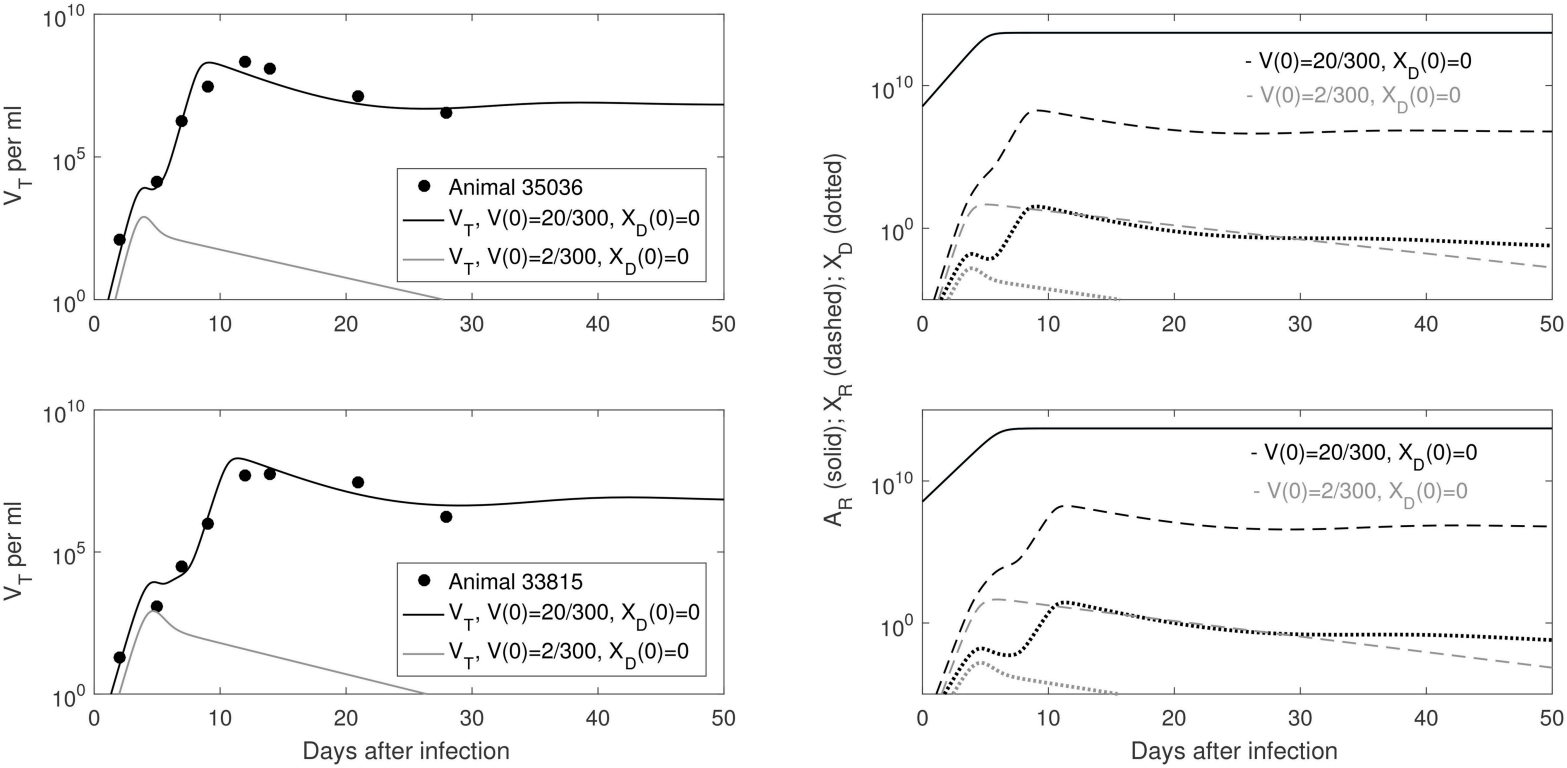
(**Left**) Basin of attraction for *V*_*T*_ given by (6) vs. data; (**Right**) Free antibody (solid lines); recipient immune complexes (dashed lines) and donor immune complexes (dotted lines) for *V*(0) = 20/300 vRNA copies per ml, *X*_*D*_(0) = 0 vRNA copies per ml (black) and *V*(0) = 2/300 vRNA copies per ml, *X*_*D*_(0) = 0 vRNA copies per ml (gray). Note that antibody populations are identical regardless of initial conditions (solid black and gray lines overlap).

Our model predicts a bistable switch in the virus dynamics between persistence and clearance when the free virus initial concentration changes from 20/300 to 2/300 copies per ml (see Figure 1, left panels). For median estimates among the two animals, we predict that for *V*(0) = 20/300 copies per ml, the total virus load increases to a maximum of 2 × 10^8^ copies per ml, before decreasing to equilibrium levels of 9 × 10^6^ copies per ml, three months after infection. By contrast, when *V*(0) = 2/300 copies per ml, the total virus concentration grows to 850 copies per ml four days following infection, before it decays below the limit of detection of 50 vRNA copies per ml.

The antibody populations do not depend of the initial viral inoculum (see Figure 1, right panels). They reach maximum carrying capacity 5 × 10^13^ molecules per ml (0.0125 mg per ml) seven days after infection. When *V*(0) = 20/300 vRNA copies per ml, free virions bind recipient antibodies to form recipient immune complexes which, at equilibrium, exceed the free virus concentration 7.8 times. When *V*(0) = 2/300 copies per ml, immune complexes are formed, but they decay below limit of detection 7 days after inoculation. Model (6) assumed that B-cell priming by the virus is followed by an antigen-independent antibody expansion with a maximum per capita growth rate *r*. We estimated a median per capita growth rate *r* = 2.14 per day, corresponding to the doubling time of antibody population of 7.8 h.

**Figure 2:**
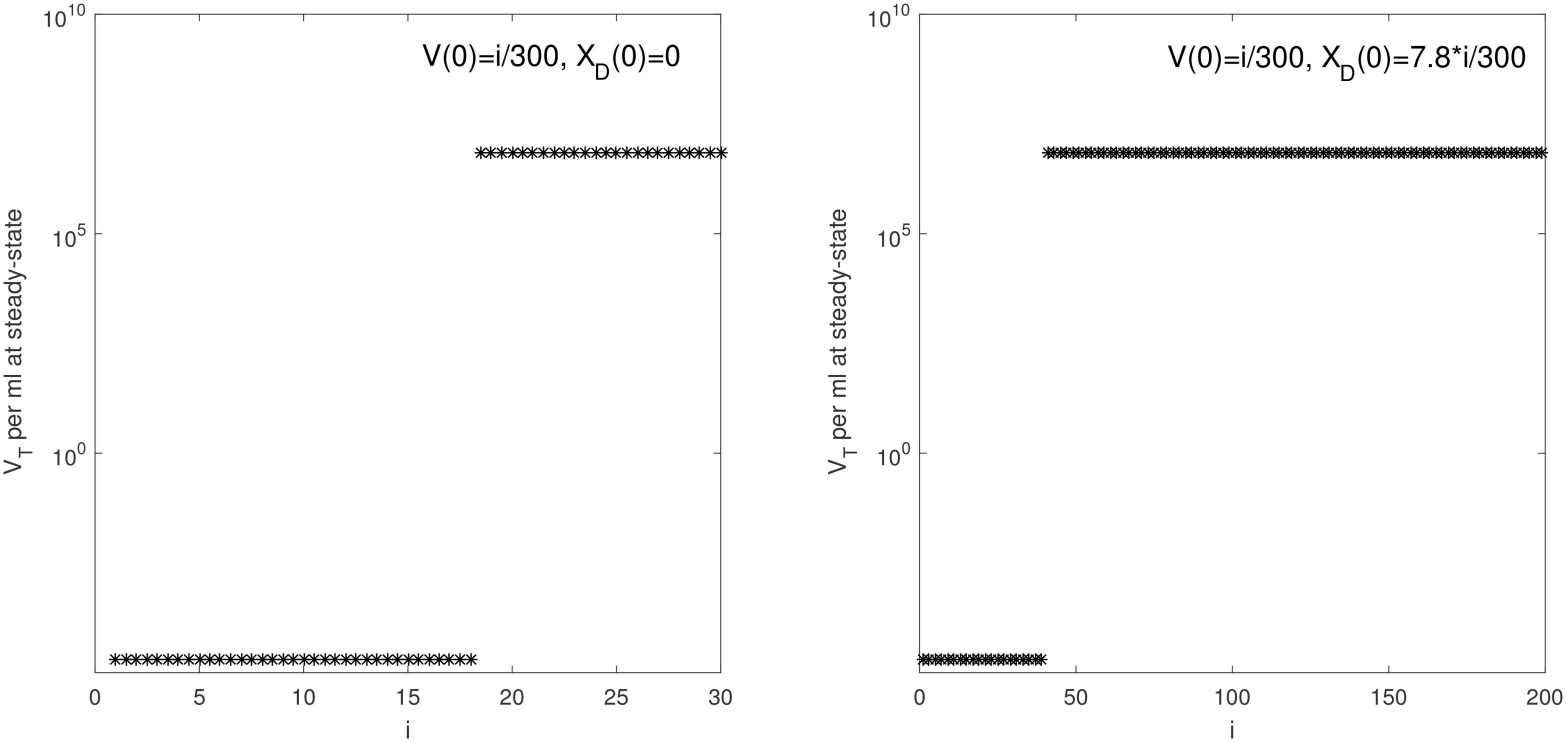
*V*_*T*_ given by (6) at steady state vs. *i* for: (**Left**) *V*(0) = *i*/300 vRNA copies per ml, *X*_*D*_(0) = 0 vRNA copies per ml and median parameters in Table 2; (**Right**) *V*(0) = *i*/300 vRNA copies per ml, *X*_*D*_(0) = 7.8 × *i*/300 vRNA copies per ml and median parameters in Table 3.

We are interested in determining the largest initial virus inoculum that allows for viral clearance under the ramp-up-stage modeling assumptions. For the median *r* parameter in Table 2 we derived a bifurcation diagram showing the asymptotic free virus concentrations three months following infection when the infectivity rate β is varied (see Figure S1). The system is displaying hysteresis. For the median infectivity rate β in Table 2, we plotted the basins of attractions for the total virus concentration *V*_*T*_ when the inoculum concentration varies (see Figure 2, left panel). We predict that *V*_*T*_ is cleared for *V*(0) < 17.5/300 copies per ml and persists otherwise.

### Infection with set-point-stage virus

Animals 33952, 34846, and 34373, were challenged with vRNA copies titrated from a plasma pool containing virus collected during the set-point-stage of infection (defined as the time several months after the peak when plasma vRNA levels were relatively stable, and antibody responses were well developed) of the seven donor monkeys (Ma et al., 2009). The animals were protected when challenged with set-point-stage plasma titrated to contain 1.5, 15, and 150 vRNA and became viremic when challenged with set-point-stage plasma containing 1500 vRNA. We aim to determine the parameter sets for which model (6) predicts a switch between viral persistence for *V*(0)+*X*_*D*_(0) = 1500/300 copies per ml and clearance for *V*(0)+*X*_*D*_(0) = 150/300 copies per ml (and consequently for 1.5/300 and 15/300 vRNA copies per ml).

As before, we assume that the donor's antibody concentration is below the limit of detection. However, there are 7.8 times more immune complexes than free virus in the inoculum plasma, *X*_*D*_(0)/*V*(0) = 7.8, as predicted by the model (6) fitted to ramp-up data and run to equilibrium values. We minimize functional *J* given by (7) with *X*_*D*_(0)/*V*(0) = 7.8. The estimated parameters are presented in Table 3.

**Table 3:** Parameter estimates and 95% confidence intervals from minimizing the likelihood function *J* given by (7) for the set-point data.

**Animal**	**β (ml day-virion^−1^)**	**95*%* CI**	***r*(day^−1^)**	**95*%* CI**	**$R_{0}^{a}$**
33952	9.3 × 10^−8^	[9.23 × 10^−8^, 9.33 × 10^−8^]	1.37	[1.369, 1.375], 0.25
34846	4.09 × 10^−8^	[4.07 × 10^−8^, 4.1 × 10^−8^]	0.515	[0.512, 0.518]	0.108
34373	1.07 × 10^−7^	[9.51 × 10^−8^, 1.07 × 10^−7^]	1.66	[1.5, 1.8]	0.2673
Median	9.3 × 10^−8^	–	1.37	–	–

**Figure 3:**
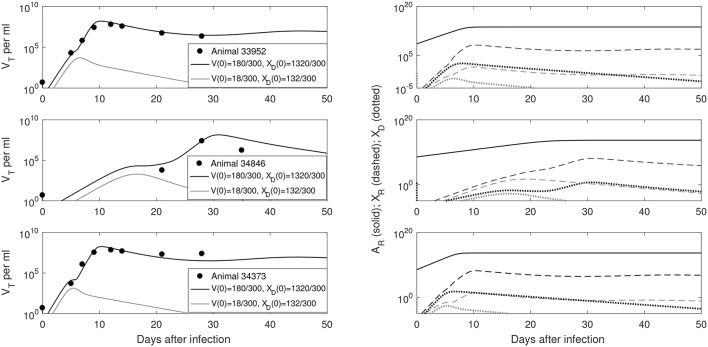
(**Left**) *V*_*T*_ given by (6) vs. data; (**Right**) Free antibody (solid lines), recipient immune complexes (dashed lines) and donor immune complexes (dotted lines): for *V*(0)+*X*_*D*_(0) = 1500/300 vRNA copies per ml, *X*_*D*_(0)/*V*(0) = 7.8 (black) and *V*(0)+*X*_*D*_(0) = 150/300 vRNA copies per ml, *X*_*D*_(0)/*V*(0) = 7.8 (gray). Note that antibody populations are identical regardless of initial conditions (solid black and gray lines overlap) but the immune complexes differ.

We predict a bistable switch between persistent and cleared virus populations based on initial conditions (see Figure 3, left panels). The median set-point virus infectivity rate is 1.5 times smaller than the median infectivity rate of the animals infected with the ramp-up-stage plasma. Moreover, the median set-point antibody antigen-dependent growth rate, *r* is 1.55 times smaller, corresponding to a median antibody population's doubling time of 12 h. We decided to compare median values, rather than averages, since the set-point plasma results are biased by the estimates in animal 34846 whose virus growth is delayed.

**Figure 4:**
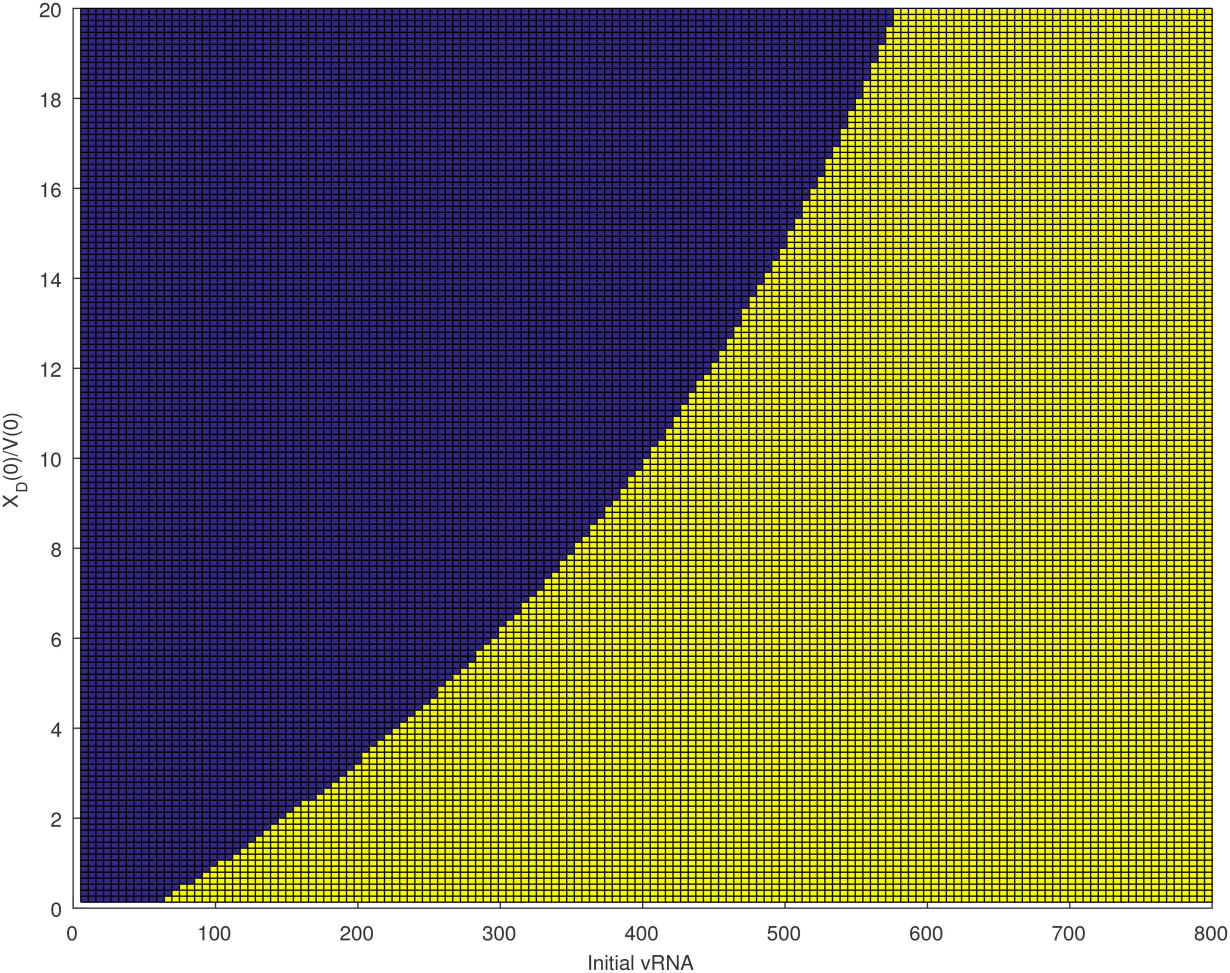
Asymptotic behavior for the *V*_*T*_ solutions of (6) for median parameters in Table 3 as the initial vRNA and *X*_*D*_(0)/*V*(0) are varied. The blue region (left of the curve) corresponds to extinction asymptotic concentrations *V*_*T*_ = 0 copies per ml. The yellow region (right of the curve) corresponds to persistence asymptotic concentrations $V_{T}=6.9\times 10^{6}$ copies per ml.

We next quantified the largest initial virus inoculum that allows for viral clearance when the initial inoculum is comprised of 7.8 times more immune complexes than free virion. We set all parameters at the median values in Table 3 and plotted the basins of attractions for the total virus concentration three months following infection as the inoculum concentration varies (see Figure 2, right panel). We predict that *V*_*T*_ is cleared when *V*_*T*_(0) ≤ 42/300 vRNA copies per ml, *X*_*D*_(0)/*V*(0) = 7.8 and persists otherwise. We also investigated the relation between viral clearance, *V*_*T*_(0) and the ratio *X*_*D*_(0)/*V*(0). We note that when the ratio between the immune complexes and free virus in the initial plasma is low, even a small inoculum size can create virus persistence. Conversely, when the immune complexes dominate the initial plasma, a large initial virus inoculum is needed to establish an infection (see Figure 4).

### Infection with a mix of heat inactivated set-point-stage plasma mixed with ramp-up-stage virions

To determine whether antibodies play a role in reducing virus infectivity, Ma et al. designed an aliquot of ramp-up stage plasma containing 20 vRNA/0.5 ml mixed with 0.5 ml of heat inactivated set-point-stage plasma (Ma et al., 2009). The set-point-stage plasma contained antibodies capable of *in vitro* neutralization. They used the aliquot on four animals: 33681, 32350, 32970, and 36068, with the first three animals being protected and the last animal developing persistent infection.

To address this experimental setting we assume the model follows the dynamics given by median parameter values in the ramp-up case with *V*(0) = 20/300 copies per ml and *X*_*D*_(0) = 0 copies per ml (see Tables 1, 2). However, we vary *A*_*D*_(0) to account for donor's antibody being present in the inoculum. We find that a minimum of $A_{D}\left(0\right)=7.4\times 10^{9}$ donor antibody molecules per ml (1.8 × 10^−6^ mg per ml) are needed for clearance. This value is above the antibody's limit of detection, but more than three orders of magnitude below the antibody's equilibrium values. This suggests that even low antibody levels may help protect the host.

**Figure 5:**
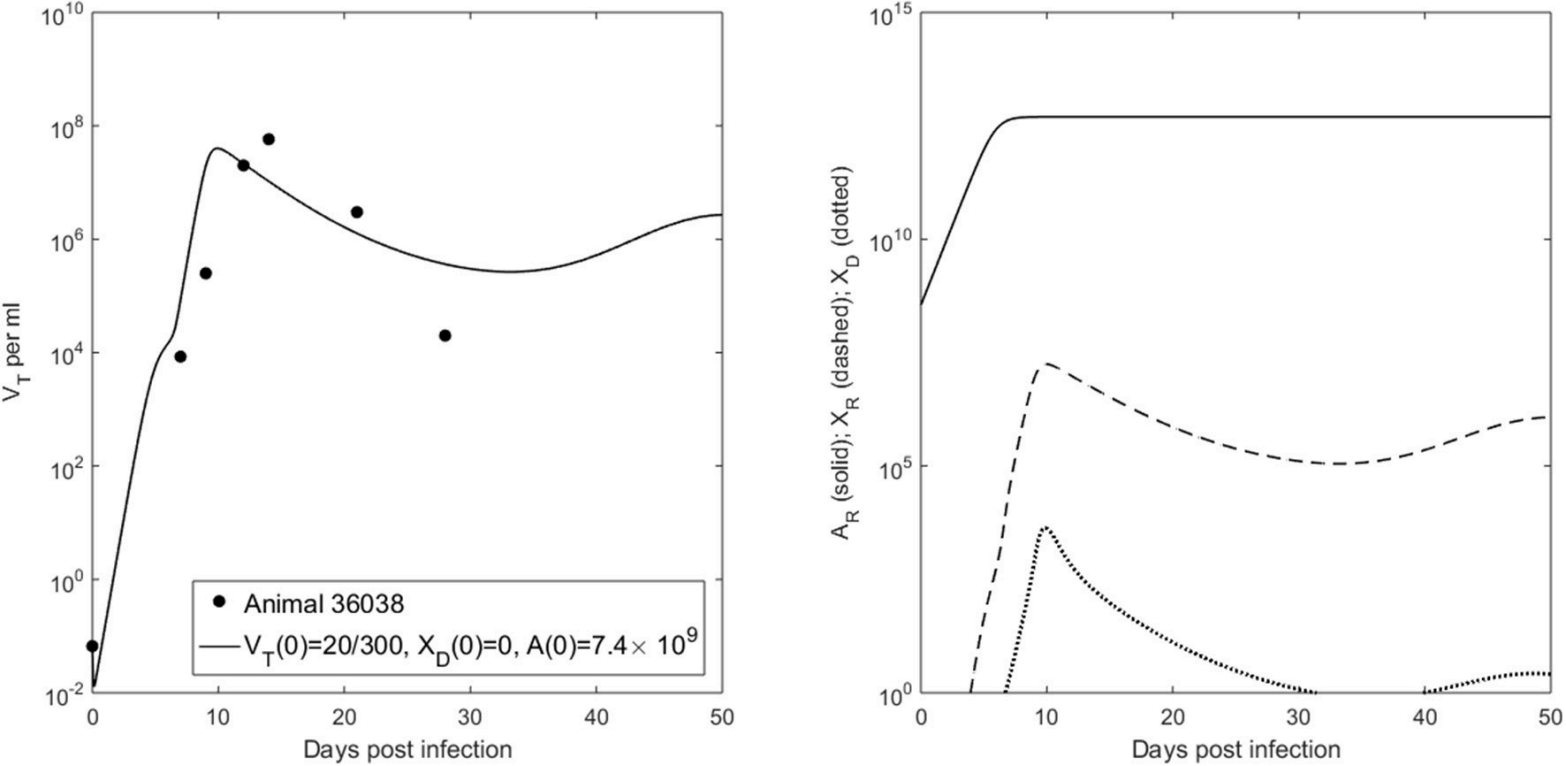
(**Left**) *V*_*T*_ given by (6) vs. data; (**Right**) Free antibody (solid lines), recipient immune complexes (dashed lines) and donor immune complexes (dotted lines) for *V*(0) = 20/300 vRNA copies per ml, *X*_*D*_(0) = 0 vRNA copies per ml and $A_{D}\left(0\right)=7.4\times 10^{9}$ molecules per ml.

We want to determine the additional recipient-virus dynamics that lead to infection in animal 36068. We fit *V*_*T*_ given by model (6) with *V*(0) = 20/300 vRNA copies per ml, *X*_*D*_(0) = 0 vRNA copies per ml and $A_{D}\left(0\right)=7.4\times 10^{9}$ molecules per ml to animal 36068's virus data and found that virus persistence is due to the recipient antibody dynamics (see Figure 5, right panel). Indeed, for a good fit we need to decrease the recipient's antibody carrying capacity to *K* = 5 × 10^12^ molecules per ml, 10 times lower than the previous carrying capacity in ramp-up infected animals. The antigen-independent expansion rate *r* = 1.62 per day is 1.3 times smaller than the median per capita antibody expansion rate in the ramp-up infected animals, but similar to that of set-point infected animals (see Table 4). This implies that animal's 36068 immune response is not strong enough to prevent persistent viremia. Moreover, for these antibody parameters bistability does not occur ($R_{0}^{a}=2.26>1$), and the virus reaches a positive steady state level regardless of the size and structure of the initial inoculum.

**Table 4:** Parameter estimates and 95% confidence intervals from fitting *V*_*T*_ to animal data for *V*(0) = 20/300 vRNA copies per ml, *X*_*D*_(0) = 0 vRNA copies per ml and $A_{D}\left(0\right)=7.4\times 10^{9}$ molecules per ml.

**Animal**	**β (ml day-virion^−1^)**	**95*%* CI**	***r*(day^−1^)**	**95*%* CI**	**$R_{0}^{a}$**
363068	1.11 × 10^−7^	[1.06 × 10^−7^, 1.17 × 10^−7^]	1.62	[1.5, 1.74]	2.26

### Can random infection and clearance events explain the data?

To address whether the switch in virus dynamics is due to random effects, we use the stochastic model of virus infection and clearance developed in Pearson et al. (2011). Under the parametrization of model (1), the *burst stochastic model* in Pearson et al. (2011), is given by

(7) $$\begin{array}{r} V\overset{\beta T}{\to }I,\\ I\overset{\delta }{\to }NV,\\ V\overset{c}{\to }\emptyset . \end{array}$$

Let *n* = (*n*_*V*_, *n*_*I*_) be the number of viruses and infected cells starting the SIV infection,

(8) $$\begin{array}{rcl} \rho_{V} & = & \mathrm{Pr}\left\{\text{Extinction}|n=\left(1,0\right)\right\}\text{and}\\ \rho_{I} & = & \mathrm{Pr}\left\{\text{Extinction}|n=\left(0,1\right)\right\}, \end{array}$$

be the probabilities of extinction given an infection that is started by one virus or one infected cell. Then the probability of virus persistence given *n*_*V*_ virion and *n*_*I*_ infected cells, $\epsilon =1-\rho_{V}^{n_{V}}\rho_{I}^{n_{I}}$, can be computed analytically. Namely

(9) $$\begin{array}{r} \begin{array}{c} \rho_{V}=\min\left\{1,\rho_{V}^{*}\right\},\\ \rho_{I}=\min\left\{1,{\left(\rho_{V}^{*}\right)}^{N}\right\}, \end{array} \end{array}$$

where $\rho_{V}^{*}$ is the positive solution of the equation

(10) $$\begin{array}{r} \gamma {\left(\rho_{V}\right)}^{N}-\rho_{V}+1-\gamma =0. \end{array}$$

**Figure 6:**
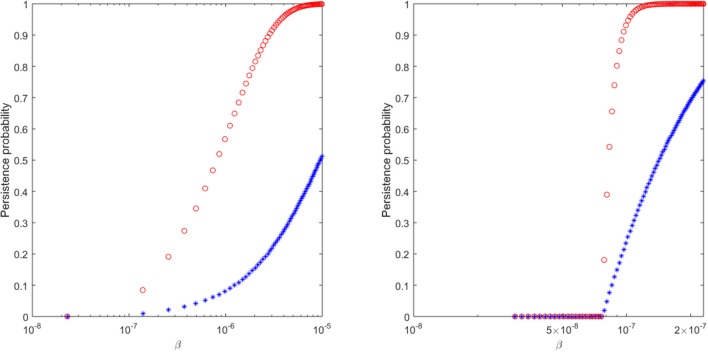
The probability of virus persistence for (**Left**) ramp-up-stage and (**Right**) set-point-stage vRNA data under the assumption of the *burst stochastic model* (9). The red circles account for the probability of persistence for high inocula, while the blue stars account for the probability of persistence for low inocula.

Note that the probability of persistence ϵ is dependent on the burst size *N* and on the probability that a virus infects a cell, $\gamma =\frac{\beta T}{\beta T+c}$ (see Pearson et al., 2011 for a full derivation). For fixed *N* = 300 virions per infected cell, *T* = 10^6^ cells per ml, and *c* = 23 per day, we determine the infectivity values β that can explain the relationship between extinction/persistence and the inoculum size in ramp-up/set-point vRNA cases when free virus establishes the infection, i.e., *n*_*I*_ = 0 (see Figure 6).

We find that a 99% probability of persistence for a ramp-up-stage inoculum of 20 vRNA occurs when the infectivity rate β>8 × 10^−6^, while the same persistence probability for a set-point inoculum of 1500 vRNA occurs for β>1.15 × 10^−7^ (see Figure 6, red circles). For these choices of β, the probability of extinction for the lower 2 ramp-up stage and 150 set-point-stage vRNA inoculum are 64% and 65%, respectively (see Figure 6, blue stars). In order for the proposed stochastic process to explain the experimental observations, there must be a 70-fold reduction in the infectivity rate β between ramp-up and set-point virus (8.75 times higher than estimated in Vaidya et al., 2010). Moreover, the extinction probability for the lower inocula is lower than ideal. While the stochastic explanation is still conceivable, we conclude that the presence of immune complexes in the inoculum is a compelling alternative explanation for the experimental outcomes.

## Discussion

We developed a mathematical model of antibody responses to SIV infection that shows a bistable switch between persistent infection and virus clearance based on the composition of the plasma used for intravenous inoculation.

We made several interesting observations. When the plasma does not contain any free donor antibody, our model predicts that the difference between infection with high inoculum containing set-point-stage virus and infection with low inoculum containing ramp-up-stage virus can be explained by the presence of immune complexes in the inoculum that exceed free virus by a factor of 7.8. Such immune complexes can still infect target CD4 T cells, but have a 10-fold decrease in infectivity. Under these assumptions, the model fits the data when the infectivity of free virus is constant over time. There is a 1.5-fold decrease in the set-point-stage free virus infectivity compared to ramp-up-stage free virus infectivity, as reported in earlier studies (Ma et al., 2009; Vaidya et al., 2010). Such decrease may be due to temporal accumulation of non-infectious particles and to the emergence of other immune factors such as CD8 T cell responses.

When the inoculum plasma contains neutralizing antibody, our model predicts that infection is blocked even when free virus infectivity rates are as high as in the ramp-up stage. In order for virus to invade, antibody responses need to be 10 times lower at equilibrium. Moreover, their growth needs 1.3-fold reduction.

We assumed that the recipient and donor antibody have high avidity of 10^8^ M^−1^ (as with infusion of broadly neutralizing antibody), a high antibody carrying capacity *K* = 5 × 10^13^ molecules per ml and estimated the recipient antibody expansion rate needed for protection. With our model, however, it is difficult to separate out the effects of changing the avidity rate *K*_*A*_ = *k*_*p*_/*k*_*m*_ and changing the antibody carrying capacity *K*. For example we can preserve the results by decreasing avidity and increasing the carrying capacity. Similarly, the antibody antigen-dependent and antigen-independent growth factors, *r* and α, have synergistic effects. If we decrease *r* we can maintain the overall virus-antibody dynamics by increasing α (as detailed in the sensitivity Figure S2).

We have also assumed that the immune complexes infect at rate β_1_ = 0.1 × β ml per day per virion. This was an arbitrary choice, and the estimated infectivity rate β may change when the β to β_1_ ratio is varied.

An important variable in our study is the basic reproduction number $R_{0}^{a}$, which has to remain below one for viral clearance. However, even when $R_{0}^{a}<1$, a persistent infection can still occur when either inoculum is composed of mostly free virion, free virus infectivity rate is high, and/or equilibrium antibody levels are low. The presence of bistable switch allows us to alter the infection outcome in the model by changing the initial antibody levels. Knowing the minimum antibody concentration needed to prevent viremia under fixed parameter setting is important for guiding vaccine design.

To address whether stochastic effects alone can explain the data, we computed the probability of virus persistence under the assumption of a *burst stochastic* model developed in Pearson et al. (2011). We find that a 70-fold decrease in virus infectivity rate β is needed between the ramp-up and set-point-inoculum in order to obtain a 99% probability of persistence for a ramp-up inoculum of 20 vRNA and a set-point inoculum of 1500 vRNA occurs. This is higher than the 8-fold decrease reported in Vaidya et al. (2010). Furthermore, the extinction probabilities for the lower ramp-up inoculum of 2 vRNA and a set-point inoculum of 150 vRNA, 64% and 65%, are lower than ideal. While the stochastic explanation is still conceivable, we conclude that the presence of immune complexes in the inoculum is a possible alternative explanation for the experimental outcome.

Our study uses data from an intravenous inoculation experiment. It is not clear if a similar relationship exist with mucosal virus inoculation or with non-neutralizing antibody responses. In fact, some antibody responses were associated with enhanced risk of heterosexual HIV acquisition in RV144. Further, immune-complexed virus may be preferentially transported across epithelial surfaces by the neonatal Fc receptor (Haynes, 2012; Gorlani and Forthal, 2013; Gupta et al., 2013, 2015). Our analysis focused only on the role of antibody in infectivity after any epithelial barriers had been crossed.

In summary, we have developed a model of virus-host dynamics during SIV infection that gives insight into the relation between the structure of infecting inoculum, virus infectivity and disease outcomes. In particular, we showed that a large set-point-stage inoculum is needed for persistent infection due to the excess of immune complexes over the free virus. Moreover, we have estimated the antibody levels and the free virus infectivity and their relation to the disease outcome when the inoculum size and structure are well understood. Such predictions are important for vaccine design.

## Author summary

We investigate whether antiviral factors can explain the change in virus infectivity during acute and chronic stages of simian immunodeficiency virus infections. Our hypothesis is that acute plasma contains mostly free virus while chronic plasma contains antibody-virus immune complexes in addition to free virus. If such immune complexes can still infect, then their infectivity rate is reduced compared to that of the free virus. We test this hypothesis by developing a mathematical model of antibody-virus dynamics that assumes interactions between virus, recipient and donor antibodies, and the corresponding immune complexes. We fit the model to published viral load data from recipient rhesus macaques challenged intravenously with different virus loads collected during donor's acute and chronic stages of SIV infection. The fits give us parameter estimates for long-run antibody concentration, free virus infectivity rate, and the relation between protection and the free virus—immune complexes ratio in the inoculum.

## Author contributions

SC and JF designed the study and performed numerical experiments. CM provided the data. SC, CM, and JF wrote the paper.

### Conflict of interest statement

The authors declare that the research was conducted in the absence of any commercial or financial relationships that could be construed as a potential conflict of interest.

## Footnotes

^1^IgG Human ELISA Kit ab100547. http://www.abcam.com/IgG-Human-ELISA-Kit-ab100547.html.
