# Supplementary material for: A Bistable Switch in Virus Dynamics Can Explain the Differences in Disease Outcome Following SIV Infections in Rhesus Macaques
Source: Front Microbiol. 2018 Jun 6;9:1216. doi: 10.3389/fmicb.2018.01216 (PMC6001289; doi:10.3389/fmicb.2018.01216)
Supplement: Supplementary file 1 [file Presentation_1.PDF]

A bi-stable switch in virus dynamics can explain the differences in disease outcome following SIV infections in rhesus macaques- Supplementary material

Section A. We study the stability conditions for the clearance steady state

$$S_0 = (s/d, 0, 0, K, 0, 0, 0).$$

The Jacobian matrix corresponding to system (4) is

$$\mathcal{J} = \begin{bmatrix} \Phi_1 & 0 & -\beta T & 0 & -\beta_1 T & 0 & -\beta_1 T \\ \Phi_2 & -\delta & \beta T & 0 & \beta_1 T & 0 & \beta_1 T \\ 0 & N\delta & \Phi_3 & -k_p V & k_m & -k_p V & k_m \\ 0 & 0 & \alpha - k_p A_R & r(1 - 2A_R/K) - k_p V & k_m & 0 & 0 \\ 0 & 0 & k_p A_R & k_p V & \Phi_4 & 0 & 0 \\ 0 & 0 & -k_p A_D & 0 & 0 & -d_A - k_p V & k_m \\ 0 & 0 & k_p A_D & 0 & 0 & k_p V & \Phi_5 \end{bmatrix},$$

where

$$\begin{aligned} \Phi_1 &= -d - \beta V - \beta_1 (X_D + X_R), \\ \Phi_2 &= \beta V + \beta_1 (X_D + X_R), \\ \Phi_3 &= -c - k_p (A_D + A_R), \\ \Phi_4 &= -k_m - c_{AV} M/(X_R + M)^2, \\ \Phi_5 &= -k_m - c_{AV} M/(X_D + M)^2. \end{aligned} \quad (1)$$

At the steady state S_0, the Jacobian reduces to

$$\mathcal{J} = \begin{bmatrix} -d & 0 & -\beta s/d & 0 & -\beta_1 s/d & 0 & -\beta_1 s/d \\ 0 & -\delta & \beta s/d & 0 & \beta_1 s/d & 0 & \beta_1 s/d \\ 0 & N\delta & -c - k_p K & 0 & k_m & 0 & k_m \\ 0 & 0 & \alpha - k_p K & -r & k_m & 0 & 0 \\ 0 & 0 & k_p K & 0 & -k_m - c_{AV}/M & 0 & 0 \\ 0 & 0 & 0 & 0 & 0 & -d_A & k_m \\ 0 & 0 & 0 & 0 & 0 & 0 & -k_m - c_{AV}/M \end{bmatrix}.$$

We have eigenvalues $\lambda_1 = -d < 0$, $\lambda_2 = -r < 0$, $\lambda_3 = -d_A$, $\lambda_4 = -k_m - c_{AV}/M$ and $\lambda_{5,6,7}$ that solve

$$\lambda^3 + Y_1 \lambda^2 + Y_2 \lambda + Y_3 = 0, \tag{2}$$

where

$$\begin{aligned} Y_1 &= \delta + c + k_p K + k_m + \frac{c_{AV}}{M}, \\ Y_2 &= c\delta - N\delta\beta s/d + (\delta + c)(k_m + \frac{c_{AV}}{M}) + k_p K(\delta + \frac{c_{AV}}{M}), \\ Y_3 &= c\delta(k_m + \frac{c_{AV}}{M} + \frac{c_{AV}}{cM} k_p K) - N\delta\beta s/d(k_m + \frac{c_{AV}}{M} + \frac{\beta_1}{\beta} k_p K). \end{aligned} \tag{3}$$

By Routh-Hurwitz conditions, $\lambda_{5,6,7}$ have negative real parts when $Y_1 > 0$, $Y_3 > 0$ and $Y_1 Y_2 - Y_3 > 0$. This happens when

$$R_0^a = R_0 \frac{k_m + \frac{c_{AV}}{M} + \frac{\beta_1}{\beta} k_p K}{k_m + \frac{c_{AV}}{M} + \frac{c_{AV}}{cM} k_p K} < 1.$$

Therefore S_0 is locally asymptotically stable when $R_0^a < 1$.

Section B: Bifurcation theory. We numerically characterize the asymptotic behavior of system (1) by deriving a bifurcation diagram, showing the asymptotic states of the free virus variable V as the infectivity rate β is changed. The system displays hysteresis, with both the extinction and persistence steady-states of free virus being possible outcomes, as the parameter β is varied back and forth (see figure S1).

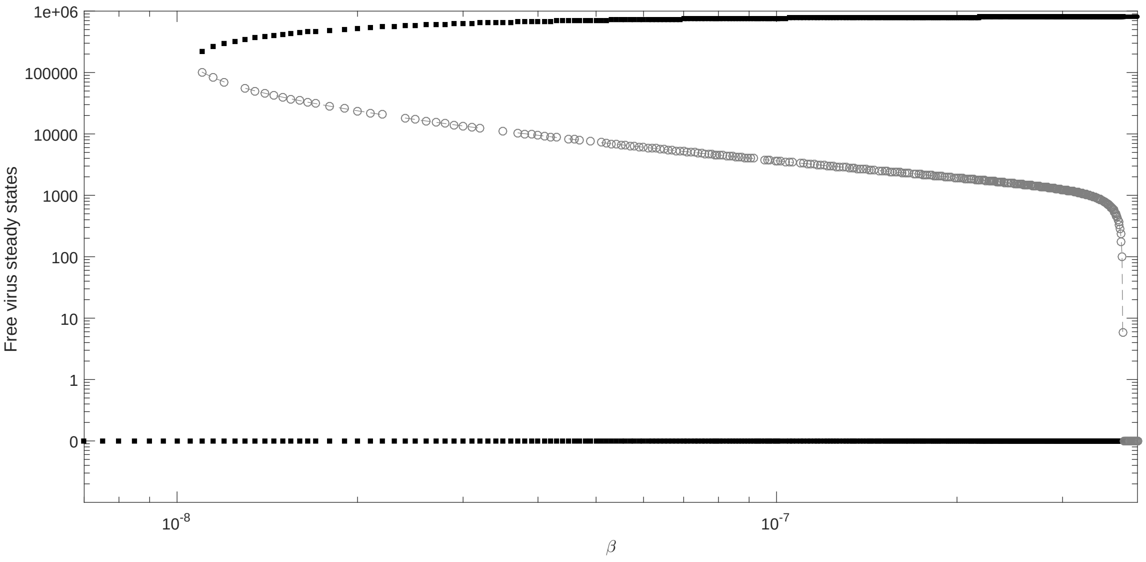

Figure S1: V given by (4) at steady state versus β. Note that the system displays hysteresis.

Section C: Sensitivity analysis. We derived the sensitivity system given by the partial derivative of variables $X = \{T, I, V, A_R, X_R, A_D, X_D\}$ as given by model (4) with respect to parameters $q = \{r, \alpha, K, k_p\}$. Details of these technique are described in [1]. The semi-relative curves $q\partial V/\partial q$ are presented in figure S2. We observe that r and α have similar effects (see figure S2, left panel). Same is true for K and k_p (see figure S2, right panel). These results show that we

cannot separate out the effects of antigen independent and antigen depended expansions. Moreover, the antibody carrying capacity and the antibody affinity have synergistic effects.

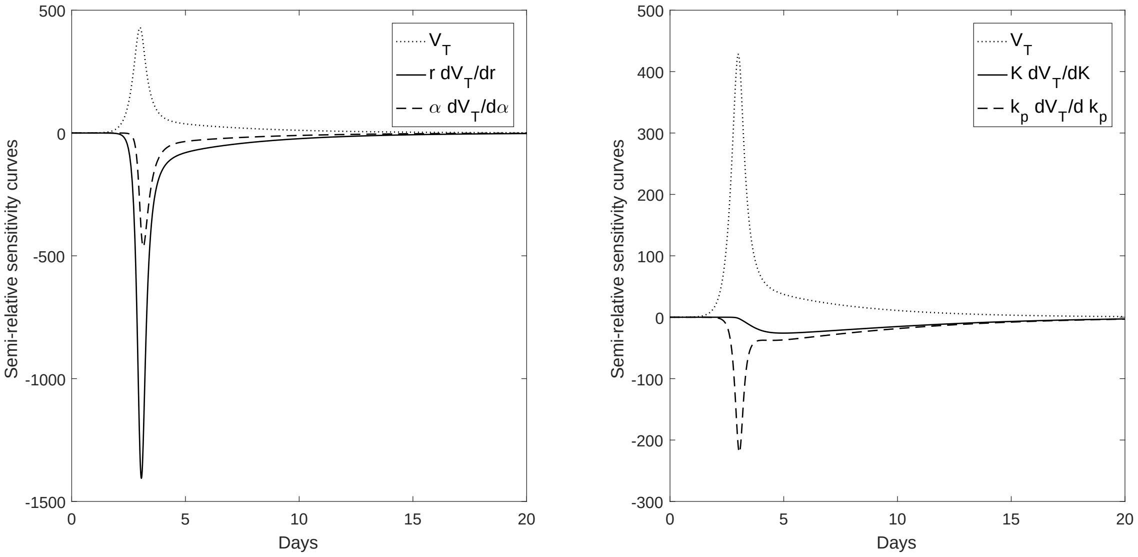

Figure S2: Semi-relative sensitivity solutions $qV_{Tq} = q\frac{\partial V_T}{\partial q}$ for: (left panel) $q = r$ (solid line), $q = \alpha$ (dashed line); (right panel) $q = K$ (solid line), $q = k_p$ (dashed line). The parameters are medians in table 2 and $\alpha = 0.01$.

References

[1] DM Bortz and PW Nelson. Sensitivity analysis of a nonlinear lumped parameter model of HIV infection dynamics. *Bull Math Biol*, 66:1009–1026, 2004.
